# What do older people value when they visit their general practitioner? A qualitative study

**DOI:** 10.1007/s10433-014-0313-0

**Published:** 2014-04-18

**Authors:** Ludmila Marcinowicz, Teresa Pawlikowska, Marek Oleszczyk

**Affiliations:** 1 Department of Family Medicine and Community Nursing, Medical University of Bialystok, Mieszka I 4 B, 15 054 Białystok, Poland; 2 Health Professions Education Centre, RCSI, Dublin, Ireland; 3 Department of Family Medicine, Jagiellonian University Medical College, Kraków, Poland

**Keywords:** Older patients, Doctor–patient relations, Quality of care, Qualitative research, Primary care

## Abstract

Older patients see their general practitioners (GPs) relatively often and so recognition of their preferences can lead to improvement of quality of care in general practice. This study aimed to identify which aspects of GPs’ behaviour are the most important for older people in their assessment of the quality of their visits and to explore the application of Jung’s taxonomy differentiating task and affective behaviour in this context. A qualitative approach to generating data was chosen. We conducted semi-structured interviews with a sample of 30 patients aged 65 and older using GP services in two demographically diverse big cities in Poland. Participants were interviewed in 2010 according to a pre-determined topic guide. This research showed that older people assess both ‘task performance’ and ‘affective performance’ behaviours of general practitioners. There were nearly twice as many patient comments concerning affective performance behaviour relative to task performance behaviour. Older people expect that their physicians will be demonstrably friendly, kind, able to joke and have enough time for the consultation.

## Introduction

An ageing population is common in European countries and is expected to escalate. In Poland, the percentage of the population aged 65 and over has grown from 10.3 % in 1990 to 13.5 % in 2009 (Central Statistical Office 2010). Older people will form an increasing group under the care of GPs. Identification of their preferences will improve quality of care in general practice.

Modern strategies to promote healthy ageing are directed, amongst others, to health care quality improvement, considering preferences and expectations of older people and feedback from the patient (Cruz-Jentoft et al. 2009). According to Coulter (2002), patient’s views on the quality of care they received should be a key part of any performance assessment in a modern health care system. Consideration of patient’s feedback supports a more patient-centred approach and brings information on ways to improve the quality of care provided (Phillips et al. 2012).

International studies show that people over 70 want to be involved in their care and perceive building a trusting relationship, having sufficient time during visits and receiving information as the central issues to involvement (Bastiaens et al. 2007). Sixma et al. (2000) developed a self-administered questionnaire for measuring quality of care from the perspective of non-institutionalised older people by using a combination of qualitative and quantitative methods. From 32 items included in the QUOTE-Elderly instrument some refer to the relation between patient and providers, for example: take patients seriously, have a friendly attitude towards the patient and develop a good understanding of the patients’ problems. Owens and Batchelor (1996) have reported very high levels of older patients’ satisfaction, but they suggested that health care professionals need to take into account the varied nature of the relationship between patient and service providers. One such context is general practice and the question is which aspect of general practitioner’s (GP’s) behaviour determines patients’ evaluations of care.

During the last decades, patients have been more frequently involved in the evaluation of health care in several countries. There are, however, scarce data available on which methods of assessment are optimal from the patient’s perspective (Bridges et al. 2008). Although quantitative techniques are widely used in research on health care, qualitative methods have recently gained increasing popularity. One reason is the variety and diversity of their results (Britten 2011). Recent reports support this view, e.g. according to Gooberman-Hill (2012) ‘There is pressing need to bridge the gap between qualitative research evidence and patient involvement in the design of research and services’.

A qualitative approach to this investigation was chosen using semi-structured interviews. This approach was employed because qualitative methods focus on people’s experiences, attitudes or beliefs and they provide a deeper understanding regarding personal topics. Britten and Fisher (1993) have suggested that general practice, like qualitative methods, involves listening to people and so becoming involved in their world. The patient’s experience of receiving care is an important part of quality assessment, and qualitative research explores which aspects of care are important to patients and, therefore, should be taken into account (Coulter 2002). Furthermore, qualitative methods can provide information to establish whether, how and to what extent older people evaluate health services (Owens and Batchelor 1996). This type of research may considerably improve our understanding of the preferences of elderly patients in relation to general practice (Berkelmans et al. 2010). The knowledge of older patients’ views and preferences is still relatively unexplored especially when derived from a qualitative approach involving older people themselves.

Jung et al. (1998) have described a taxonomy for categorising physician behaviour in consultations based on work by Hall et al. (1988) (Table 1). The authors conducted semi-structured interviews with patients using the ‘critical incident technique’. This technique described by Flanagan (1954) focuses on obtaining a record of specific behaviours that have actually happened (incidents) rather than general opinions. Using this technique, Jung et al. (1998) described seventeen mutually exclusive categories of general practitioner behaviour divided into two dimensions: task performance and affective performance.

**Table 1:** Comparison of physician behaviour categorisation

Jung et al. (1998)	Hall et al. (1988)
Task performance	
Information giving	Information giving
General	General
Drugs and treatment	Drugs and treatment
Examination	Examination
Illness	Illness
Questions	Questions
	General
	Closed questions
	Open questions
	Compliance monitoring
Action	Competence
	Technical
	Interpersonal
Medical-technical competence	
General	
Burden on patient	
Affective performance	
Socio-emotional behaviour	Socio-emotional behaviour
Body movements	Body movements
Social conversation	Social conversation
Understanding	Positive talk
Support	Negative talk
Enough time	
Friendliness	
Partnership building	Partnership building
General	
Continuity	
Participating questions	
Other	
Not applicable	
Does not feel competent to judge	

The aim of the study was: (1) to identify which aspects of GPs’ behaviour are the most important for older people in their perception of the quality of the GP visits and (2) to explore the application of Jung’s taxonomy differentiating task and affective behaviour in this context.

## Methods

### Sample

The recruitment procedure focused on patients aged 65 and older using GP services in two demographically diverse big cities in Poland Bialystok and Krakow. Study participants were selected from eight different GP surgeries. This was designed to capture the diversity of genuine experiences of care provided by GPs. A maximum variety sample was chosen purposively to achieve a study group of various ages, genders, education and health problems (Patton 2002). Study participants were selected by the first author in cooperation with GPs.

The inclusion criteria were age (65 and over), health status allowing participation in the interview, and patients’ willingness and consent to participate in the study. After obtaining informed consent from participants, the researcher made appointments to interview them individually. Patient interviews continued until no new themes emerged, and finally 30 individuals aged 65–87 years were interviewed.

### Data collection

Semi-structured interviews were conducted by the first author (LM) in 2010 according to a pre-determined topic guide (Table 2). Probes were open-ended and patients were encouraged to give free expression to identify which aspects of GP’s behaviour are the most important for older people in general practice when they assess quality of care of the aged. Interviews lasted 0.5–2 h and were usually undertaken at patients’ homes (the exceptions: four interviews were conducted in a university building and one at a participant’s workplace, at their request). All interviews were tape-recorded and fully transcribed by LM.

**Table 2:** Topic guide for interviews

Thinking back to your most recent consultation
In your opinion, what was the most important in the GP consultation?
What does it mean to you to be satisfied with a GP consultation?
Which of your GP’s behaviours did you like?
Which of your GP’s behaviours did you dislike?

The study was approved by the Ethics Committee of the Medical University of Bialystok, Poland (R-I-002/251/2009).

### Data analysis

The transcripts of interviews were read through several times and then analysed thematically with regard to patients’ perception of GP’s behaviour. First, all statements related to GP’s behaviour were noted using standard manual qualitative techniques of open coding. The second stage of analysis involved grouping 12 mutually exclusive categories of GPs’ behaviour which had been noted into two dimensions: ‘task performance’ and ‘affective performance’. This categorisation was based upon Jung’s taxonomy (1996). Two authors (LM and TP) analysed the data independently and a consensus on categorisation was achieved through mutual discussion (Barbour 2001). Any discrepancies were resolved through discussion with a medical sociologist. All statements were grouped in terms of positive and negative affective performance. Illustrative quotes are used in this paper. The problem of quality in qualitative health research is widely discussed in literature (Mays and Pope 1995; Barbour 2001). As Britten and Fisher (1993) have pointed out, it might be true that ‘quantitative methods are reliable but not valid and that qualitative methods are valid but not reliable’.

## Results

Characteristics of participants are illustrated in Table 3. From a total of 30 participants, 18 were female and 12 were male and their mean age was 74 (range 65–87).

**Table 3:** Participants’ characteristics

Characteristics	*n* = 30
Mean age (range)	74 (65–87)
Age	
65–74	16
75–87	14
Gender	
Female	18
Male	12
Education	
Elementary	7
Technical	10
Secondary	7
University	6

### Preferred GP’s behaviours

In total, 122 patient comments regarding GP behaviour were identified. Of these, 42 remarks were regarding ‘task behaviours’ and 76 concerned ‘affective behaviours’ (4: ‘other’). Although both positive and negative comments were elicited, the majority was positive—101 and 21 negative (Table 4).

**Table 4:** Physician behaviour reported by participants (*n* = 122 comments from 30 patients)

Performance	Positive	Negative	Total
	*n* = 101	*n* = 21	*n* = 122
Task performance	31	11	42
Information giving	10	4	14
Questions	3	3	6
Action	10	3	13
Medical-technical competence	8	1	9
Affective performance	66	10	76
Socio-emotional behaviour	64	7	71
Body movements (8)			
Social conversation (11)			
Understanding (14)			
Support (7)			
Enough time (9)			
Friendliness (22)			
Partnership building	2	3	5
Other	4	–	4

### Task performance

In this dimension, we grouped the following categories: Information giving, Questions, Action and Medical-technical competence.

#### Information giving

Fourteen participants valued GP’s behaviour with respect to information giving. Patients valued doctors giving explanations and advice which could be general or more detailed e.g.

“He explained to me what this cholesterol is about” (Woman, aged 67).

In contrast patients were especially negative regarding the lack of information: “He just prescribes something and says nothing, explains nothing” (Man, aged 66).

#### Questions

In 6 participants’ opinion direct questioning by the GP (‘How do you feel?’ ‘What happened?’ ‘Does it hurt?’) during the consultation was crucial in terms of encouragement.

“He asks me, what is the matter with me? How do I feel” (Man, aged 73).

This contrasts with negative experiences where participants suggested, how GPs should behave, e.g.

“She *may* ask me, if I take my drugs regularly (…). She *should* ask me, how I feel after those drugs” (Woman, aged 79).

Some patients want to have the chance to express their opinions and feelings during the consultation.

“If I was a doctor, I would ask—‘How do you feel’? What is the matter with you?’ But doctors would just repeat the drug prescription and ask for how long. And good-bye. Even they wouldn’t check my blood pressure, nothing. (…) I am not stupid, I’ve experienced some things in my life, too” (Woman, aged 71).

Other participants cared about being interviewed in detail by GP about their complaints, fearing that they [the patient] might omit or forget something important e.g.

“One should ask, pump a little bit from the patient, not just rely on what patient said, because he [the patient] cannot say everything, he might be nervous, he might be not able to name his disease, because it is difficult, very difficult” (Woman, aged 76).

#### Action

In this category, GPs’ behaviours referred to actions e.g. referring for additional tests, referring to specialists, taking blood pressure and examining (auscultation). Participants’ perceptions—both positive and negative—were precise and practical:

“Somehow doctor was not very eager to give a referral for tests” (Woman, aged 79).

“He measured my blood pressure, examined me” (Man, aged 72).

#### Medical-technical competence

Some participants mentioned those GP competences which allow them to diagnose and treat effectively, but these statements were often quite general:

“He can treat, he can give such and such a drug, which helps” (Man, aged 73).

“My GP knows my disease and prescribes good drugs” (Woman, aged 76).

### Affective performance

In this dimension, the following categories were included: Socio-emotional behaviour (body movements, social conversation, understanding, support, enough time and friendliness) and partnership building.

#### Body movements

Participants paid attention to such GP behaviours as shaking hands, pointing out the place where the patient can sit, looking at the patient and smiling:

“When I enter the surgery, she is smiling, she points her hand—please be seated” (Woman, aged 68).

“Suddenly she turns to the computer, I mean she turns away her attention, because I don’t know what she is reading there, if a drug is needed or some record-keeping” (Man, aged 80).

#### Social conversation

Participants liked it when GPs used their first name, and valued being asked about other family members or chatting about topics viewed as mutual to establish a connection, e.g.

“The doctor calls me ‘Mr. Wiktor’, because I often come here. Already I am satisfied with this!” (Man, aged 81).

“Even to talk about the fact that doctor and I, we both live in the same quarter” (Man, aged 80).

#### Understanding

Participants also paid attention to such GP behaviours as listening, empathy and interest in patient’s problems.

“I like it, that he is a doctor who always likes to listen to patient” (Woman, aged 74).

“He shows his concern as I would be his family member, although: I think he wouldn’t have to do this. With this he impressed me” (Man, aged 82).

#### Support

Positive behaviours, as perceived by patients, were often reported as being most important for them:

“When the GP would look at you kindly, talk to you, cheer you up and even say—please, do not worry, it will be all right” (Woman, aged 71).

Statements of some participants clearly showed how they valued the healing power of consultation with the doctor.

“He would advise in illness in such a way that you would leave satisfied. Maybe this disease is growing, but if you talk to him you are leaving healthier (…). They cure even with words” (Man, aged 75).

#### Enough time

Some of the participants focused on time spent on the visit, e.g.

“She has time for patient always, she would talk, she would ask, she is not on hurry, because someone else is waiting. And at this age, it is known, we want to talk to someone” (Woman, aged 66). Contrasting with evident haste “I have a female doctor, who is also a medical director of this health care center, she’s so hurried. There’s a visit, and she is running out of the surgery” (Man, aged 80).

#### Friendliness

A large number of the participants’ comments (all positive) were dedicated to overt friendliness during conversation. Participants said that they could be relaxed during consultations. They observed whether the GPs’ behaviour was kind, friendly and pleasant.

“Young, elegant, nice; with a smile and sense of humor she treats her patients” (Man, aged 82).

“This doctor is very nice. She is so family-orientated, truly she is a family doctor. I like her very much” (Man, aged 73).

#### Partnership building

In this category, participants’ comments concerned two issues: cooperation in decisions concerning patient’s health and continuity of care through contact with the same GP.

“We were thinking together, what to do in my situation; it was a relaxed conversation” (Man, aged 75). Contrasting with “He [doctor] should know his patients” (Woman aged 77).

### Doctor-centric views

Other statements were categorised as relating to doctor-centric or hierarchical world views.

“He knows what he does; he is an educated doctor and he knows how to treat people” (Woman aged 72).

“I myself do not like to assess people (it’s the doctor’s job)” (Man, aged 82).

Interestingly, a participant who had completed higher education and was professionally active stated:

“I think that older people should be treated a little bit like children (…) An aged person needs their doctor to take care of her” (Woman aged 76).

## Discussion

Older people assess both ‘task performance’ and ‘affective performance’ behaviours; however, they pay more attention to ‘affective performance’ behaviour and perceive it more positively. Their opinion of ‘task performance’ is voiced at a somewhat general level and is more critical. If there is a complaint by older patients, it concerns insufficient or even lacking information on their health status from GPs. During consultations older people pay more attention to socio-emotional behaviours of doctors, especially to ‘friendliness’ and other behaviours which further engagement such as joking or ‘social conversation'.

Jung's et al. (1998) taxonomy for categorising physician behaviour was a useful approach to structuring our results and deepening our understanding of the perception of older people in consultations with GPs. The results reveal that when patients describe GPs’ behaviours which they value in consultations, their perception relates mainly to the socio-emotional behaviour of doctors. Our study confirms that during the visit patients pay attention both to verbal and non-verbal communication (Marcinowicz et al. 2010; Pawlikowska et al. 2012; Robben et al. 2012) and they want a patient-centred and positive approach (Little et al. 2001).

Although physicians’ behaviours reported by participants may be formally categorised, the content of patients’ statements should also be carefully considered and analysed as these statements provide valuable information. Some of the doctor’s socio-emotional behaviours seem to be particularly important as they might influence patients’ satisfaction with the consultation as well as a subjective feeling of healthiness. For instance, some participants’ quotes demonstrate that a GP visit itself is therapeutic: ‘He would advise in illness in such a way that you would leave satisfied. Maybe this disease is growing, but if you talk to him you are leaving healthier (…). They cure even with words’ (Man, aged 75). More specifically, our findings highlight the importance of good communication for patients of 65 years and older.

There are two reasons which make the patients’ statements categorised as ‘doctor–centric’ in our study important. First, older patients in this category are relatively unwilling to assess health care providers, which is consistent with other studies showing that older people are reluctant to articulate their dissatisfaction (Owens and Batchelor 1996). Second, our findings suggest that some patients actually prefer the paternalistic model of a physician–patient relationship which had been predominant in Poland. This is also indicated by the low number of comments which related to partnership building. The results of the *European telephone survey* show that a relatively high proportion of Polish patients felt that the doctor, rather than the patient, should be the primary decision maker. About 60 % of Polish respondents felt the patient should have a role in treatment decisions, either sharing responsibility for decision making with the doctor or being the primary decision maker, compared with 91 % of Swiss respondents and 87 % of German respondents. Moreover, this contrasts with a consistent trend which has been reported for younger people to want a more patient-centred approach in comparison to older people (Coulter and Jenkinson 2005).

In qualitative research, an important issue is to achieve credibility and to identify all views and opinions that may shed different but relevant light on the issue studied (Graneheim and Lundman 2004). Choosing participants with various experiences and from different GP settings increases the trustworthiness of the findings.

This study demonstrates some important considerations for achieving a better understanding of older patients’ evaluations of general practice care. Physicians’ awareness of their own behaviours during consultations can considerably influence GPs’ attitudes and their decisions on how to deliver the best health care for older patients.

## Conclusions

Above all, older people value socio-emotional behaviours of GPs in consultations. They expect that their physicians will be demonstrably friendly, kind, able to joke and have enough time for the consultation. Effective realisation of these behaviours is important for a better understanding of the way patients evaluate primary care contacts and to improve quality of care for older people.
